# COVID-19 and children returning to school

**DOI:** 10.1016/j.bjorl.2020.12.010

**Published:** 2021-01-19

**Authors:** Rujittika Mungmunpuntipantip, Viroj Wiwanitkit

**Affiliations:** a Private Academic Consultant, Bangkok, Thailand; b Dr DY Patil University, Pune, India

Dear Editor,

We would like to share ideas on the report “COVID-19 in children: considerations for returning to school”.^1^ Guimarães et al. concluded that “*We believe we must inform our patients of the real situation and health risks that children and adolescents will face with the reopening of schools, consider the losses caused by the lack of school for them and ….*”.^1^ To manage the outbreak, the lockdown process is usually required but there must be the good local area control. Regarding school closure, it is approved for usefulness in reducing of incidence of COVID-19 as well as other respiratory virus infection in the pediatric group.^2^ Nevertheless, the school closure has to be concomitantly used with other preventive measures, such as office closures.^3^ If there is only a school closure without other measures, the children might wander around during the holidays and it might result in more difficulty in blocking of COVID-19 spreading area.^3^ The holistic focus on the whole community system is required in controlling of COVID-19 outbreak.^3^

## Conflicts of interest

The authors declare no conflicts of interest.
